# Predictors of guideline-concordant treatment intensification in metastatic castration-sensitive prostate cancer: an explainable machine learning study

**DOI:** 10.3389/fonc.2026.1835503

**Published:** 2026-10-13

**Authors:** Emmanuel Barton Odro, James Bychinski, Ted Van Alstyne, Connor Corsini, Parker Owen, Cheryl Page Jones, Nicole Krebs, Benjamin Solomon, Ryan Vaca, Casey Williams, Rachel Elsey, Tobias Meissner

**Affiliations:** 1 Avera Cancer Institute, Sioux Falls, SD, United States; 2 Pfizer Inc, Groton, NY, United States; 3 University of South Dakota, Sanford School of Medicine, Sioux Falls, SD, United States

**Keywords:** guideline-concordant care, healthcare access, machine learning, metastatic castration-sensitive prostate cancer, novel hormonal therapy, real-world evidence, SHAP, treatment intensification

## Abstract

**Background:**

Treatment intensification with androgen deprivation therapy (ADT) combined with docetaxel, an androgen receptor pathway inhibitor, or selected triplet regimens is recommended for eligible patients with metastatic castration-sensitive prostate cancer (mCSPC). Despite increasing adoption, implementation remains variable in routine clinical practice.

**Objective:**

To characterize temporal trends in first-line systemic treatment for mCSPC and identify factors associated with receipt of guideline-concordant treatment intensification using exploratory explainable machine learning.

**Methods:**

National treatment patterns were evaluated using the Pfizer Oncology Quality Care Insight Tool derived from IQVIA Anonymized Patient Longitudinal Data, and institutional patterns were evaluated within Avera Health. The exploratory cohort included 42 patients with documented mCSPC treated between 2018 and 2023. Guideline-concordant treatment intensification was defined as ADT combined with docetaxel or a novel hormonal therapy within 90 days of ADT initiation. Potential outcome-derived variables were excluded before modeling. Six predictive approaches were evaluated using repeated nested stratified cross-validation, with 5-fold outer cross-validation repeated 20 times and 5-fold inner cross-validation repeated three times. Recursive feature elimination was performed within outer training folds, and class imbalance was addressed using SMOTE within resampling.

**Results:**

Thirty-two (32) of forty-two (42) patients (76.2%) received guideline-concordant treatment intensification. Treatment-pattern analysis showed increasing use of first-line novel hormonal therapy and declining ADT monotherapy over time. Under repeated nested validation, discrimination was modest and variable across models. The Decision Tree achieved the highest patient-level AUC (0.675; 95% CI, 0.421–0.929), whereas Random Forest achieved an AUC of 0.650 (95% CI, 0.383–0.917), accuracy of 85.7%, precision of 88.2%, recall of 93.8%, and F1 score of 0.909. Feature-selection stability identified insurance coverage for novel hormonal therapy as the most consistently selected predictor, followed by positive core proportion and smoking history. These three variables were retained in the final explanatory Random Forest model, and global SHAP and Random Forest importance analysis showed broadly concordant rankings.

**Conclusions:**

Treatment intensification for mCSPC increased substantially in routine practice. Repeated nested validation produced more conservative and variable performance estimates than the original single-split analysis, underscoring the exploratory nature of this small cohort. Healthcare access and selected clinical factors warrant further evaluation in larger, multicenter datasets.

## Introduction

Metastatic castration-sensitive prostate cancer (mCSPC) represents a critical stage in the management of advanced prostate cancer, during which early treatment decisions substantially influence long-term clinical outcomes. Over the past decade, multiple randomized clinical trials have demonstrated that treatment intensification with androgen deprivation therapy (ADT) combined with docetaxel, androgen receptor pathway inhibitors—including abiraterone, enzalutamide, apalutamide, and darolutamide—or selected triplet therapy regimens significantly improves overall survival compared with ADT alone. These findings have established treatment intensification as the recommended standard of care for eligible patients with mCSPC and have been incorporated into contemporary international clinical practice guidelines (1–8).

The expanding availability of effective systemic therapies has transformed the treatment landscape for mCSPC. Real-world studies have demonstrated increasing adoption of novel hormonal therapy (NHT)-based treatment strategies, accompanied by a decline in ADT monotherapy. Nevertheless, implementation of guideline-concordant treatment remains inconsistent across healthcare systems, institutions, and patient populations. Population-based analysis continue to demonstrate substantial variation in the use of treatment intensification, suggesting that many eligible patients do not receive evidence-based therapy despite well-established survival benefits (9–16).

Treatment selection in mCSPC is inherently multifactorial. In addition to tumour burden, metastatic presentation, comorbidity, and functional status, clinicians must consider patient preferences, anticipated treatment-related toxicities, financial barriers, insurance coverage, medication access, and logistical considerations. Several observational studies have further demonstrated that socioeconomic characteristics and healthcare access contribute to disparities in receipt of intensified treatment, indicating that non-biological factors influence treatment decisions in routine oncology practice (17, 18).

Traditional statistical approaches, including multivariable regression, have provided important insights into factors associated with treatment selection but generally assume linear relationships and limited interactions among predictors. Machine learning (ML) offers a complementary analytical framework capable of identifying complex nonlinear relationships and higher-order interactions within routinely collected clinical data. However, many ML algorithms function as “black-box” models, limiting clinical interpretability. Explainability methods such as SHapley Additive exPlanations (SHAP) help address this limitation by quantifying the contribution of individual predictors to model predictions, thereby facilitating clinical interpretation while preserving predictive flexibility (19, 20).

The primary objective of this study was to characterize temporal trends in first-line systemic therapy for patients with mCSPC using national and institutional real-world treatment data. The secondary objective was to identify clinical and healthcare-related factors associated with receipt of guideline-concordant treatment intensification using an exploratory explainable machine learning framework. Given the modest sample size, the machine learning analysis were designed to be hypothesis-generating rather than predictive for clinical implementation. To improve methodological rigor, model development incorporated repeated nested cross-validation, recursive feature elimination performed within training folds, and *post hoc* explainability analysis to identify stable predictors associated with treatment intensification in routine clinical practice.

## Materials and methods

### Study design and setting

This retrospective observational study evaluated temporal patterns of first-line systemic therapy for metastatic castration-sensitive prostate cancer (mCSPC) and explored clinical and healthcare-related factors associated with receipt of guideline-concordant treatment intensification. The study incorporated two complementary data sources. National treatment patterns were characterized using the Pfizer Oncology Quality Care Insight Tool (QCIT), which is derived from IQVIA Anonymized Patient Longitudinal Data. Institutional treatment patterns and the exploratory predictive analysis were based on electronic health record data from Avera Health (21).

The institutional cohort included patients treated between 2018 and 2023 who met prespecified eligibility criteria for mCSPC. The machine learning component was designed as an exploratory, hypothesis-generating analysis rather than as a clinical prediction tool because of the limited cohort size.

### Identification of the mCSPC cohort

Metastatic castration-sensitive prostate cancer was identified through detailed review of the electronic health record, including clinician documentation, prostate cancer history, metastatic disease status, and systemic treatment history. Patients were eligible if metastatic prostate cancer was documented while the disease remained castration-sensitive at cohort entry. Patients with documented metastatic castration-resistant prostate cancer or localized prostate cancer at cohort entry were excluded.

This approach was used because mCSPC status was determined clinically from the longitudinal medical record rather than from a single diagnosis code or isolated laboratory measurement.

Baseline demographic and clinical characteristics according to receipt of guideline-concordant treatment intensification are summarized in **Table 1**.

**Table 1:** Baseline demographic and clinical characteristics according to receipt of guideline-concordant treatment intensification

Characteristic	Overall (N=42)	Not guideline-concordant (n=10)	Guideline-concordant (n=32)
Ethnicity, n (%)			
Hispanic or Latino	1 (2.4%)	1 (10.0%)	0 (0.0%)
Not Hispanic or Latino	41 (97.6%)	9 (90.0%)	32 (100.0%)
Race, n (%)			
African American	2 (4.8%)	0 (0.0%)	2 (6.2%)
Caucasian	39 (92.9%)	10 (100.0%)	29 (90.6%)
Native American	1 (2.4%)	0 (0.0%)	1 (3.1%)
Age at cancer diagnosis, years			
Mean (SD)	69.24 (9.00)	69.90 (10.43)	69.03 (8.68)
Median [IQR]	66.50 [64.00, 75.00]	70.00 [63.25, 78.00]	66.50 [64.00, 74.25]
Age at metastatic diagnosis, years			
Mean (SD)	71.79 (8.52)	72.30 (9.64)	71.62 (8.30)
Median [IQR]	71.00 [66.25, 78.00]	73.00 [66.75, 79.75]	71.00 [66.50, 77.25]
Charlson Comorbidity Index			
Mean (SD)	2.62 (1.15)	2.80 (1.32)	2.56 (1.11)
Median [IQR]	2.00 [2.00, 3.00]	2.00 [2.00, 3.00]	2.00 [2.00, 3.00]
Elixhauser Comorbidity Index			
Mean (SD)	6.12 (4.52)	7.00 (5.54)	5.84 (4.22)
Median [IQR]	4.00 [4.00, 9.00]	4.00 [4.00, 9.75]	4.00 [4.00, 9.00]
Pretreatment PSA level			
Mean (SD)	246.40 (500.71)	93.36 (122.27)	294.22 (563.31)
Median [IQR]	19.86 [5.38, 130.50]	29.74 [7.85, 128.65]	19.86 [4.80, 136.97]
Gleason score			
Mean (SD)	8.50 (1.02)	8.40 (1.17)	8.53 (0.98)
Median [IQR]	9.00 [7.25, 9.00]	8.50 [7.25, 9.00]	9.00 [7.75, 9.00]
Positive core proportion			
Mean (SD)	0.65 (0.29)	0.64 (0.33)	0.66 (0.28)
Median [IQR]	0.65 [0.38, 0.92]	0.72 [0.28, 0.95]	0.62 [0.42, 0.92]
ECOG			
Mean (SD)	0.48 (0.67)	0.50 (0.71)	0.47 (0.67)
Median [IQR]	0.00 [0.00, 1.00]	0.00 [0.00, 1.00]	0.00 [0.00, 1.00]
Tumor burden, n (%)			
High	20 (47.6%)	6 (60.0%)	14 (43.8%)
Low	22 (52.4%)	4 (40.0%)	18 (56.2%)
Metastatic status, n (%)			
De novo	21 (50.0%)	4 (40.0%)	17 (53.1%)
Relapsed	21 (50.0%)	6 (60.0%)	15 (46.9%)
Insurance coverage for NHT, n (%)			
No	7 (16.7%)	6 (60.0%)	1 (3.1%)
Yes	35 (83.3%)	4 (40.0%)	31 (96.9%)
Genetic mutation, n (%)			
No	24 (57.1%)	7 (70.0%)	17 (53.1%)
Yes	18 (42.9%)	3 (30.0%)	15 (46.9%)
Pathogenic mutation, n (%)			
No	39 (92.9%)	10 (100.0%)	29 (90.6%)
Yes	3 (7.1%)	0 (0.0%)	3 (9.4%)
Patient area type, n (%)			
Rural	19 (45.2%)	5 (50.0%)	14 (43.8%)
Urban	23 (54.8%)	5 (50.0%)	18 (56.2%)
State, n (%)			
Iowa	2 (4.8%)	2 (20.0%)	0 (0.0%)
Minnesota	5 (11.9%)	1 (10.0%)	4 (12.5%)
North Dakota	1 (2.4%)	0 (0.0%)	1 (3.1%)
Nebraska	1 (2.4%)	1 (10.0%)	0 (0.0%)
South Dakota	33 (78.6%)	6 (60.0%)	27 (84.4%)
Marital status, n (%)			
Divorced	3 (7.1%)	0 (0.0%)	3 (9.4%)
Life Partner	1 (2.4%)	0 (0.0%)	1 (3.1%)
Married	29 (69.0%)	8 (80.0%)	21 (65.6%)
Single	4 (9.5%)	1 (10.0%)	3 (9.4%)
Widow/Widower	5 (11.9%)	1 (10.0%)	4 (12.5%)
Employment status, n (%)			
Full-Time Employed	5 (11.9%)	2 (20.0%)	3 (9.4%)
Part-Time Employed	1 (2.4%)	0 (0.0%)	1 (3.1%)
Retired	32 (76.2%)	7 (70.0%)	25 (78.1%)
Self-Employed	4 (9.5%)	1 (10.0%)	3 (9.4%)
Insured, n (%)			
No	14 (33.3%)	3 (30.0%)	11 (34.4%)
Yes	28 (66.7%)	7 (70.0%)	21 (65.6%)
Smoking history, n (%)			
No	22 (52.4%)	6 (60.0%)	16 (50.0%)
Yes	20 (47.6%)	4 (40.0%)	16 (50.0%)

ECOG, Eastern Cooperative Oncology Group; IQR, interquartile range; NHT, neoadjuvant hormonal therapy; PSA, prostate-specific antigen; SD, standard deviation.

### Study outcome

The primary outcome for the exploratory predictive analysis was receipt of guideline-concordant treatment intensification. Treatment intensification was defined as initiation of androgen deprivation therapy (ADT) in combination with docetaxel or a novel hormonal therapy (NHT), including abiraterone, enzalutamide, apalutamide, or darolutamide, within 90 days of ADT initiation.

The 90-day interval was selected to capture treatment intensification occurring as part of the initial mCSPC management strategy while allowing for real-world delays related to oncology referral, diagnostic workup, treatment planning, insurance authorization, medication access, and coordination between urology and medical oncology. A similar 90-day operational definition has been used in previously published real-world research evaluating treatment intensification in mCSPC (22).

Patients who did not receive qualifying intensified therapy within the specified interval were classified as not having received guideline-concordant treatment intensification.

Because the available dataset did not permit reliable reconstruction of alternative treatment windows for all patients, sensitivity analysis using 60-day, 120-day, or 180-day definitions were not performed.

### Candidate predictors

Candidate variables represented demographic, clinical, disease-related, comorbidity, healthcare-access, and geographic characteristics available in the electronic health record. Variables considered included age at prostate cancer diagnosis, age at metastatic diagnosis, race, ethnicity, Charlson Comorbidity Index, Elixhauser Comorbidity Index, pretreatment prostate-specific antigen level, Gleason score, positive core proportion, Eastern Cooperative Oncology Group performance status, tumor burden, metastatic presentation, genetic and pathogenic mutation status, insurance status, insurance coverage for NHT, patient area type, state of residence, marital status, employment status, and smoking history.

Variables with substantial missingness that were not considered sufficiently reliable for modeling, including alcohol use and frontier status, were excluded before imputation and model development.

Two additional variables, NHT receipt and NHT assistance needed, were excluded from the candidate predictor set before feature selection and model development. NHT receipt directly overlapped with the treatment-intensification outcome definition, and NHT assistance need could arise downstream of the decision to prescribe treatment. Their exclusion was intended to reduce the potential for outcome leakage or incorporation of post-treatment information into model development.

Insurance coverage for NHT was retained in the primary model because it represented a healthcare-access characteristic rather than treatment receipt itself. Its influence was evaluated separately in a sensitivity analysis excluding this variable.

### Missing data

Missingness was assessed for all candidate variables before preprocessing. Variables with substantial missingness judged unsuitable for reliable modeling were excluded. Remaining missing values were imputed using k-nearest-neighbor imputation with (k=5).

Missingness was reassessed after imputation to confirm completion of the analytic dataset. Tables describing variable-level missingness before and after imputation were generated to provide transparency regarding data completeness and preprocessing.

Because the present analysis was exploratory and based on a small cohort, imputation was used to preserve available observations rather than excluding patients through complete-case analysis.

### Class distribution and class-imbalance handling

The final analytic cohort included 42 patients, of whom 32 (76.2%) received guideline-concordant treatment intensification and 10 (23.8%) did not.

Because of the imbalance between outcome classes, synthetic minority oversampling was applied using the Synthetic Minority Over-sampling Technique (SMOTE). Importantly, synthetic observations were generated only within model-training resamples and were not created before partitioning the data. Consequently, observations in outer validation folds remained untouched by the balancing procedure.

This approach was selected in preference to simple duplication-based random upsampling to reduce the risk that repeated copies of minority-class observations would contribute to overfitting in the small dataset.

### Feature assessment and selection

Information Gain was calculated for candidate predictors as a descriptive assessment of their univariate information content with respect to the treatment-intensification outcome. Information Gain was not used as an independent screening threshold for inclusion in the final models.

Formal feature selection was performed using recursive feature elimination (RFE) with Random Forest-based ranking. To reduce optimistic bias, RFE was performed separately within each outer training sample during nested cross-validation rather than on the complete dataset before validation.

Candidate feature-set sizes of 3, 5, 7, 9, 11, 13, and 15 predictors were evaluated when permitted by the number of available predictors in the relevant training sample. Feature-selection stability was subsequently quantified as the proportion of outer validation resamples in which each predictor was selected.

Accordingly, Information Gain, RFE, and SHAP served distinct purposes: Information Gain provided descriptive univariate ranking, RFE was used for multivariable feature selection during model development, and SHAP was used only after model fitting to interpret model predictions.

### Model development

Six predictive approaches were evaluated:

1. Logistic Regression
2. Decision Tree
3. Neural Network
4. Random Forest
5. Support Vector Machine with radial basis kernel
6. A stacked ensemble combining Support Vector Machine, Neural Network, and Random Forest predictions

Categorical variables were converted to indicator variables using model-matrix encoding. Predictor levels were defined consistently between training and validation samples, and zero-variance predictors were removed within the corresponding training data.

Random Forest models used 500 trees. Model-specific hyperparameters were optimized within the inner resampling procedure. For the stacked model, predictions generated within the inner training resamples from the Support Vector Machine, Neural Network, and Random Forest models were combined using logistic regression as the meta-learner.

A classification probability threshold of 0.50 was used for calculation of binary classification metrics.

### Repeated nested cross-validation

Because of the small sample size and to reduce dependence on a single random train-test split, model development and performance estimation were conducted using repeated nested stratified cross-validation.

The outer validation procedure consisted of 5-fold stratified cross-validation repeated 20 times, resulting in 100 outer validation resamples. Each outer training sample was used for feature selection, class balancing, hyperparameter tuning, and model fitting, while the corresponding outer fold was reserved exclusively for performance evaluation.

Within each outer training sample, model tuning used 5-fold cross-validation repeated three times, subject to the number of minority-class observations available in that training sample. SMOTE was applied only within these model-training resamples.

This design ensured that feature selection, class balancing, and hyperparameter optimization were conducted independently of the corresponding outer validation observations.

### Model performance

Model performance was evaluated using accuracy, precision, recall (sensitivity), specificity, F1 score, and area under the receiver operating characteristic curve (AUC).

Performance was summarized in two complementary ways. First, metrics were calculated separately for each of the 100 outer validation resamples to characterize the variability of model performance. For each metric, the mean, standard deviation, median, and 2.5th and 97.5th percentiles across outer resamples were calculated.

Second, because each patient contributed out-of-fold predictions across repeated cross-validation iterations, predicted probabilities were averaged at the patient level for each model. These repeated out-of-fold average probabilities were used to generate the final ROC curves and patient-level performance estimates. DeLong 95% confidence intervals were calculated for patient-level AUC estimates.

Given the small cohort and substantial overlap in model-performance estimates, differences between models were interpreted descriptively rather than as evidence of statistically established superiority.

### Model explainability

Random Forest was selected for explainability analysis because it provided a clinically interpretable framework for evaluating nonlinear predictor contributions while maintaining favorable classification performance. Its use for explainability was distinct from selection of the model with the numerically highest AUC.

After completion of repeated nested validation, RFE was repeated on the complete leakage-safe analytic dataset to identify the predictors used in the final explanatory Random Forest. This final model was fitted for interpretation only and was not used as the source of the reported validation performance estimates.

SHapley Additive exPlanations (SHAP) were calculated to quantify the contribution of individual predictors to Random Forest predictions. Global importance was summarized using mean absolute SHAP values across patients. A local SHAP analysis was also generated to illustrate predictor contributions for an individual patient.

Traditional Random Forest variable importance was additionally calculated and compared with the global SHAP ranking as a complementary assessment of predictor importance.

### Sensitivity analysis

Because insurance coverage for NHT represented a potentially influential healthcare-access factor, a prespecified sensitivity analysis was conducted excluding this variable.

The Random Forest analysis was repeated using the same repeated nested cross-validation framework, including RFE within outer training samples and SMOTE within model-training resamples. Performance after removal of insurance coverage was compared with the corresponding primary Random Forest results to assess the extent to which predictive performance depended on this access-related variable.

### Treatment-pattern analysis

Annual first-line treatment patterns were summarized descriptively for the national QCIT population and the Avera Health population. Treatment categories included NHT-based therapy, first-generation antiandrogen-based therapy, luteinizing hormone-releasing hormone monotherapy, chemotherapy-based treatment, and NHT plus chemotherapy.

National treatment patterns were evaluated from 2018 through 2024, whereas Avera Health treatment patterns were evaluated from 2018 through 2023. Annual proportions were displayed using stacked bar charts to characterize changes in treatment adoption over time.

### Statistical software and reproducibility

All analysis were performed in R version 4.3.3 (23). The analytic workflow was implemented as a single reproducible script incorporating preprocessing, imputation, feature assessment, nested cross-validation, model development, model evaluation, explainability analysis, sensitivity analysis, and generation of manuscript figures.

Random seeds were prespecified to support reproducibility. The final reproducibility workflow also recorded R package versions, session information, analysis settings, and a checksum of the input analytic dataset. Publication figures were generated programmatically and exported as high-resolution TIFF files.

## Results

### Cohort characteristics

The final analytic cohort consisted of 42 patients with documented metastatic castration-sensitive prostate cancer who met all eligibility criteria. Of these, 32 patients (76.2%) received guideline-concordant treatment intensification, whereas 10 patients (23.8%) did not receive intensified treatment within 90 days of androgen deprivation therapy initiation. Variables with substantial missingness (alcohol use and frontier status) were excluded before preprocessing, and the remaining missing values were successfully addressed using k-nearest-neighbor imputation. Two variables, NHT receipt and NHT assistance needed, were excluded before model development because of their potential to introduce outcome leakage. Information Gain rankings for the leakage-safe predictor set are presented in **Supplementary Table 2**.

### National and institutional treatment patterns

National treatment patterns demonstrated progressive adoption of first-line novel hormonal therapy (NHT)-based treatment between 2018 and 2024, accompanied by a gradual decline in androgen deprivation therapy (ADT) monotherapy and first-generation antiandrogen-based treatment (**Figure 1**). The proportion of patients receiving NHT-based treatment increased from 27% in 2018 to 46% in 2024, whereas ADT monotherapy declined modestly from 41% to 38% over the same period.

**Figure 1:**
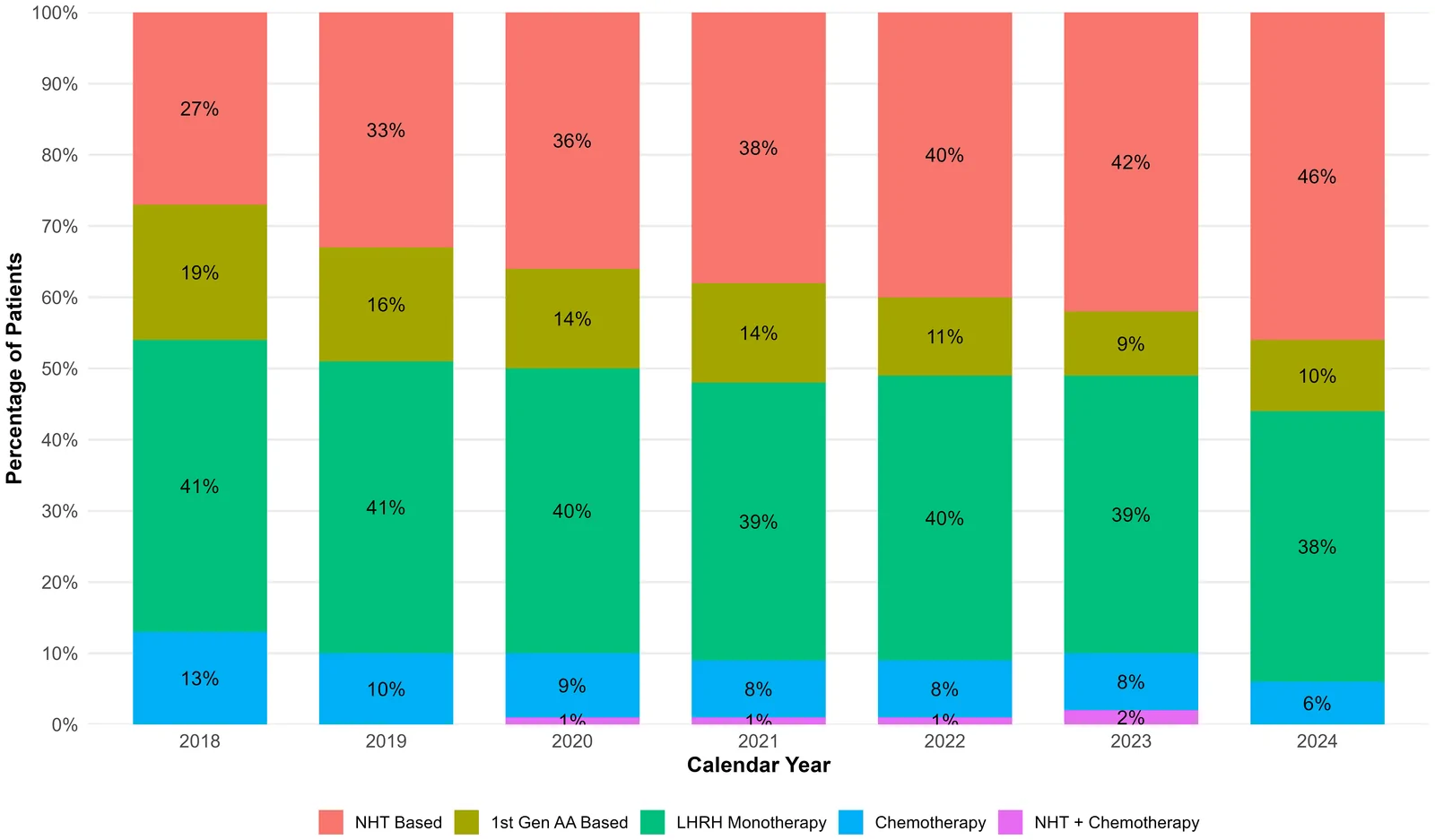
First-line treatment trends for newly diagnosed mCSPC patients at the National level.

Institutional treatment patterns at Avera Health showed a similar but more pronounced trend (**Figure 2**). NHT-based treatment increased from 8% in 2018 to 88% in 2023, with corresponding reductions in ADT monotherapy and first-generation antiandrogen-based treatment. These findings demonstrate progressive implementation of contemporary treatment recommendations at both the national and institutional levels.

**Figure 2:**
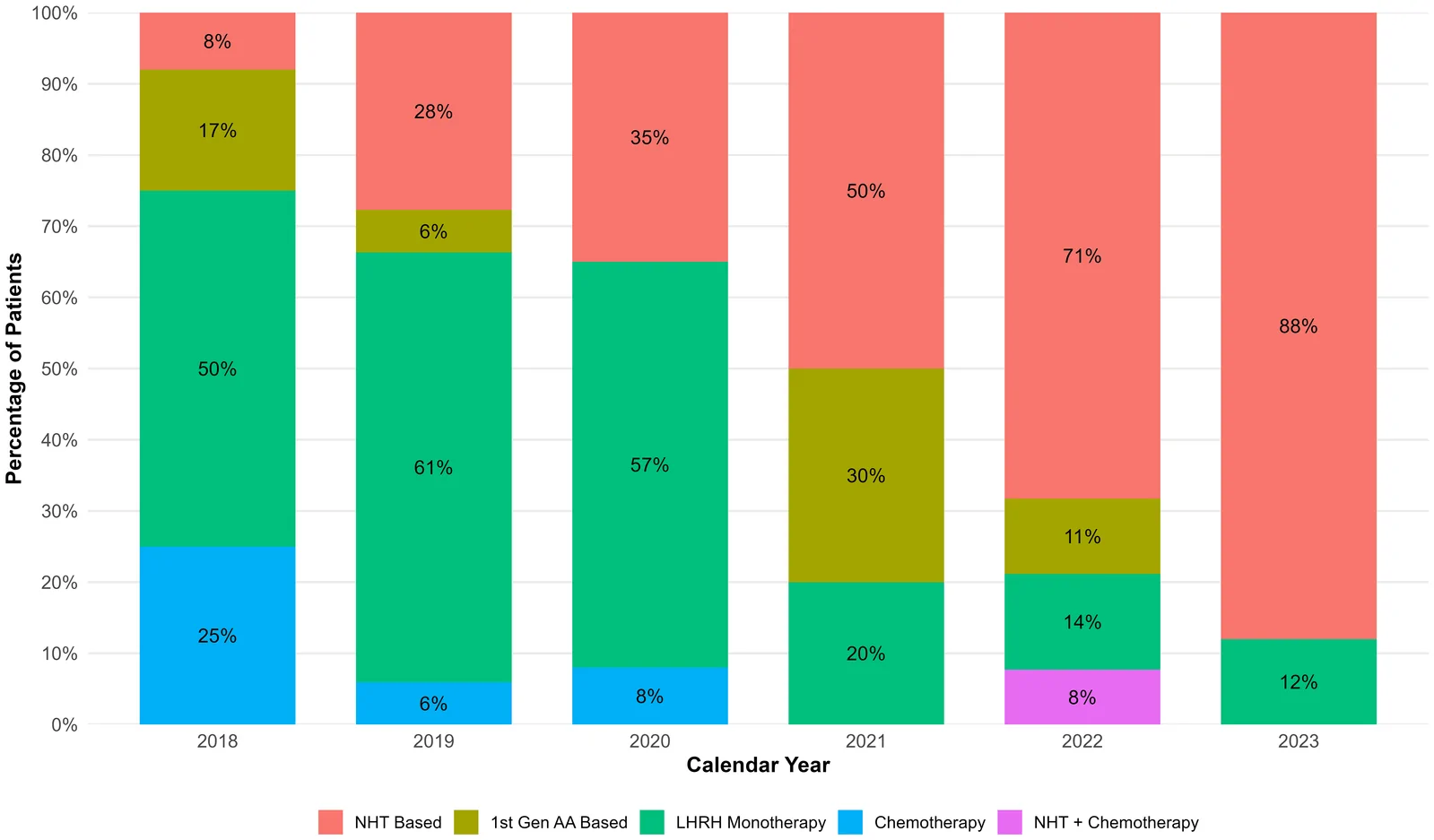
First-line treatment trends for newly diagnosed mCSPC patients at Avera Health.

### Repeated nested cross-validation performance

Six predictive approaches were evaluated using repeated nested stratified cross-validation consisting of 5-fold outer cross-validation repeated 20 times, with 5-fold inner cross-validation repeated three times for model tuning.

Across the 100 outer validation resamples, discrimination varied considerably between resamples, reflecting the modest sample size. The Decision Tree demonstrated the highest mean discrimination during repeated validation, followed by Random Forest, whereas the remaining models showed comparable but slightly lower performance. Fold-level performance distributions are summarized in **Table 2**.

**Table 2:** Model performance distributions across repeated nested cross-validation resamples.

Model	Metric	Mean	SD	Median
Decision Tree	Accuracy	0.769	0.147	0.778
	Precision	0.856	0.095	0.857
	Recall	0.848	0.183	0.857
	Specificity	0.515	0.329	0.500
	F1 score	0.839	0.118	0.857
	AUC	0.709	0.186	0.732
Logistic Regression	Accuracy	0.779	0.150	0.778
	Precision	0.851	0.104	0.857
	Recall	0.870	0.161	1.000
	Specificity	0.490	0.362	0.500
	F1 score	0.852	0.111	0.857
	AUC	0.631	0.272	0.616
Neural Network	Accuracy	0.751	0.159	0.750
	Precision	0.840	0.101	0.833
	Recall	0.838	0.205	0.857
	Specificity	0.470	0.347	0.500
	F1 score	0.831	0.130	0.857
	AUC	0.654	0.236	0.667
Random Forest	Accuracy	0.741	0.154	0.778
	Precision	0.837	0.105	0.857
	Recall	0.828	0.192	0.857
	Specificity	0.465	0.335	0.500
	F1 score	0.819	0.136	0.857
	AUC	0.735	0.212	0.750
Support Vector Machine	Accuracy	0.777	0.145	0.778
	Precision	0.859	0.100	0.857
	Recall	0.855	0.154	0.857
	Specificity	0.530	0.339	0.500
	F1 score	0.849	0.107	0.857
	AUC	0.757	0.218	0.786
Stacked Model	Accuracy	0.753	0.155	0.778
	Precision	0.829	0.107	0.833
	Recall	0.857	0.173	0.857
	Specificity	0.420	0.353	0.500
	F1 score	0.834	0.120	0.857
	AUC	0.647	0.249	0.655

Patient-level performance estimates derived from repeated out-of-fold predictions are presented in **Table 3**. The Decision Tree achieved the highest patient-level area under the receiver operating characteristic curve (AUC) of 0.675 (95% CI, 0.421–0.929). Random Forest achieved an AUC of 0.650 (95% CI, 0.383–0.917), with an accuracy of 85.7%, precision of 88.2%, recall of 93.8%, specificity of 60.0%, and an F1 score of 0.909. Logistic Regression, Neural Network, Support Vector Machine, and the stacked ensemble demonstrated similar overall classification performance, although all models exhibited wide confidence intervals consistent with the exploratory nature of the cohort.

**Table 3:** Patient-level model performance using averaged repeated out-of-fold predictions.

Model	Accuracy	Precision	Recall	Specificity	F1 score	AUC (95% CI)
Decision Tree	0.833	0.857	0.938	0.500	0.896	0.650 (0.395–0.905)
Logistic Regression	0.833	0.857	0.938	0.500	0.896	0.619 (0.330–0.908)
Neural Network	0.833	0.857	0.938	0.500	0.896	0.669 (0.422–0.916)
Random Forest	0.833	0.857	0.938	0.500	0.896	0.722 (0.505–0.939)
Support Vector Machine	0.833	0.879	0.906	0.600	0.892	0.769 (0.563–0.975)
Stacked Model	0.833	0.857	0.938	0.500	0.896	0.681 (0.447–0.916)

AUC, area under the receiver operating characteristic curve; CI, confidence interval; SVM, support vector machine.

Receiver operating characteristic curves for all evaluated models are shown in **Figure 3**, illustrating overlapping discrimination between algorithms and emphasizing the variability expected in a relatively small dataset. Calibration of the Random Forest model using repeated out-of-fold patient-level probabilities is presented in **Figure 4**.

**Figure 3:**
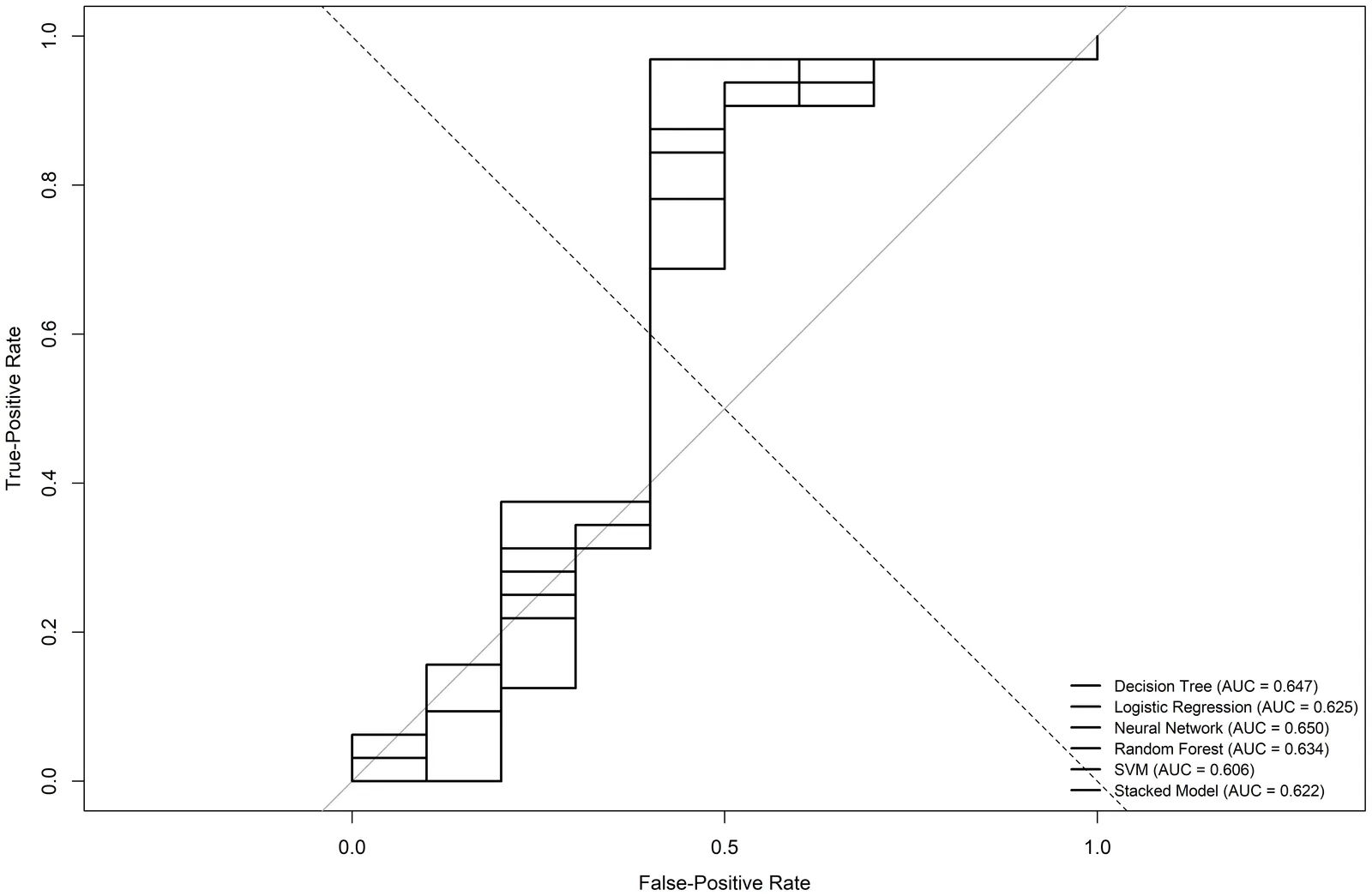
Receiver Operating Characteristic (ROC) curves for the evaluated predictive models.

**Figure 4:**
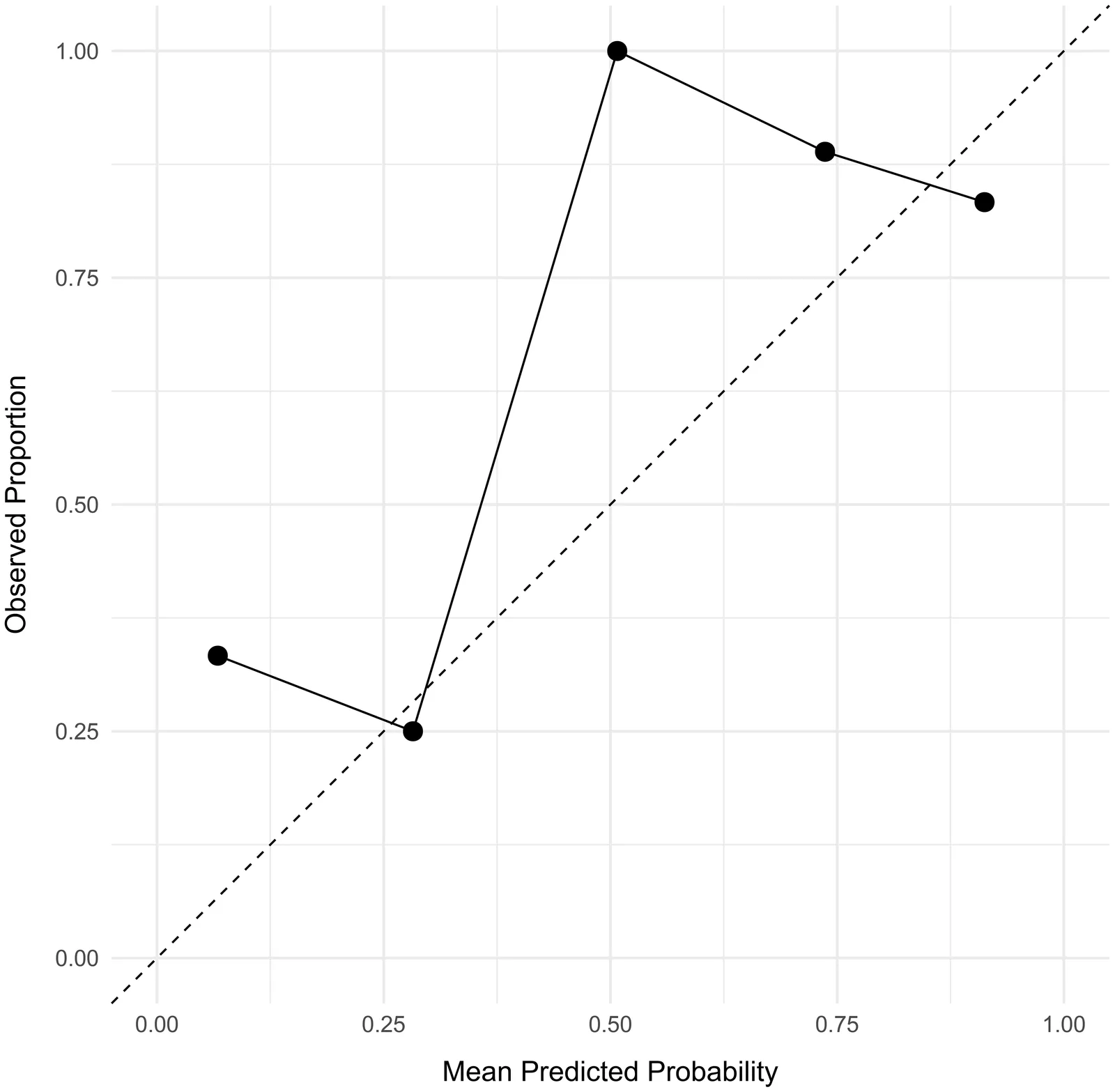
Calibration of the random forest model using repeated nested cross-validation.

### Feature selection stability

Recursive feature elimination was performed independently within each outer training sample to reduce optimistic bias. Feature-selection stability was subsequently evaluated across all outer validation resamples.

Insurance coverage for novel hormonal therapy was selected in 100% of outer validation resamples, representing the most consistently retained predictor. Other frequently selected variables included positive core proportion (71%), state of residence (46%), and smoking history (45%). The remaining predictors were selected less consistently, indicating greater uncertainty regarding their contribution across repeated resampling.

When recursive feature elimination was repeated using the complete leakage-safe analytic dataset for the final explanatory model, three predictors were retained: insurance coverage for novel hormonal therapy, positive core proportion, and smoking history.

### Explainable machine learning

Because Random Forest demonstrated favorable classification performance while providing a flexible framework for model interpretation, it was selected for explainability analysis following completion of repeated nested cross-validation.

Global SHAP analysis identified insurance coverage for novel hormonal therapy as the most influential predictor of treatment intensification, followed by smoking history and positive core proportion (**Figure 5**). Local SHAP analysis illustrated how these predictors contributed to the predicted probability of treatment intensification for an individual patient (**Figure 6**).

**Figure 5:**
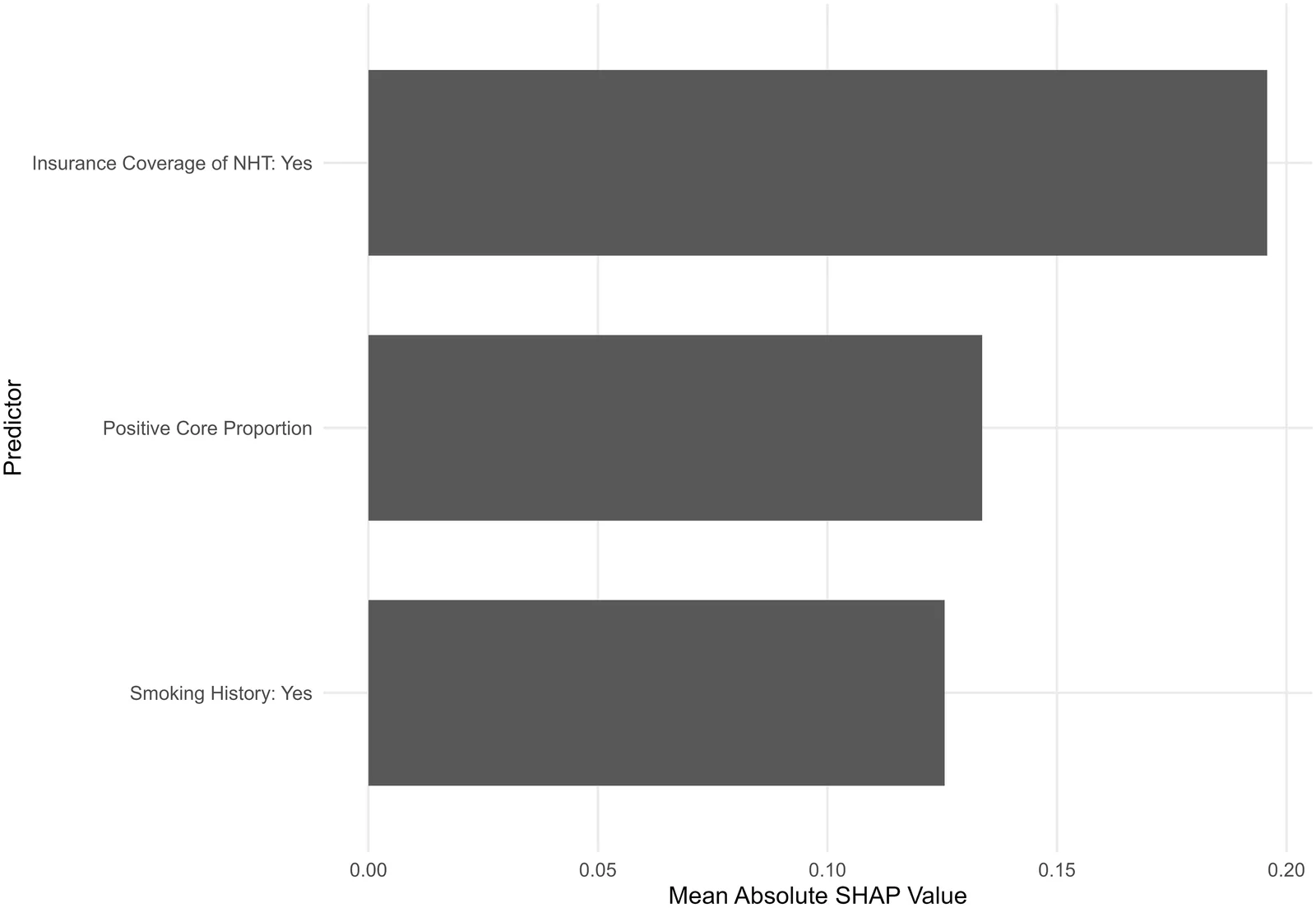
Global SHAP feature importance for the random forest model.

**Figure 6:**
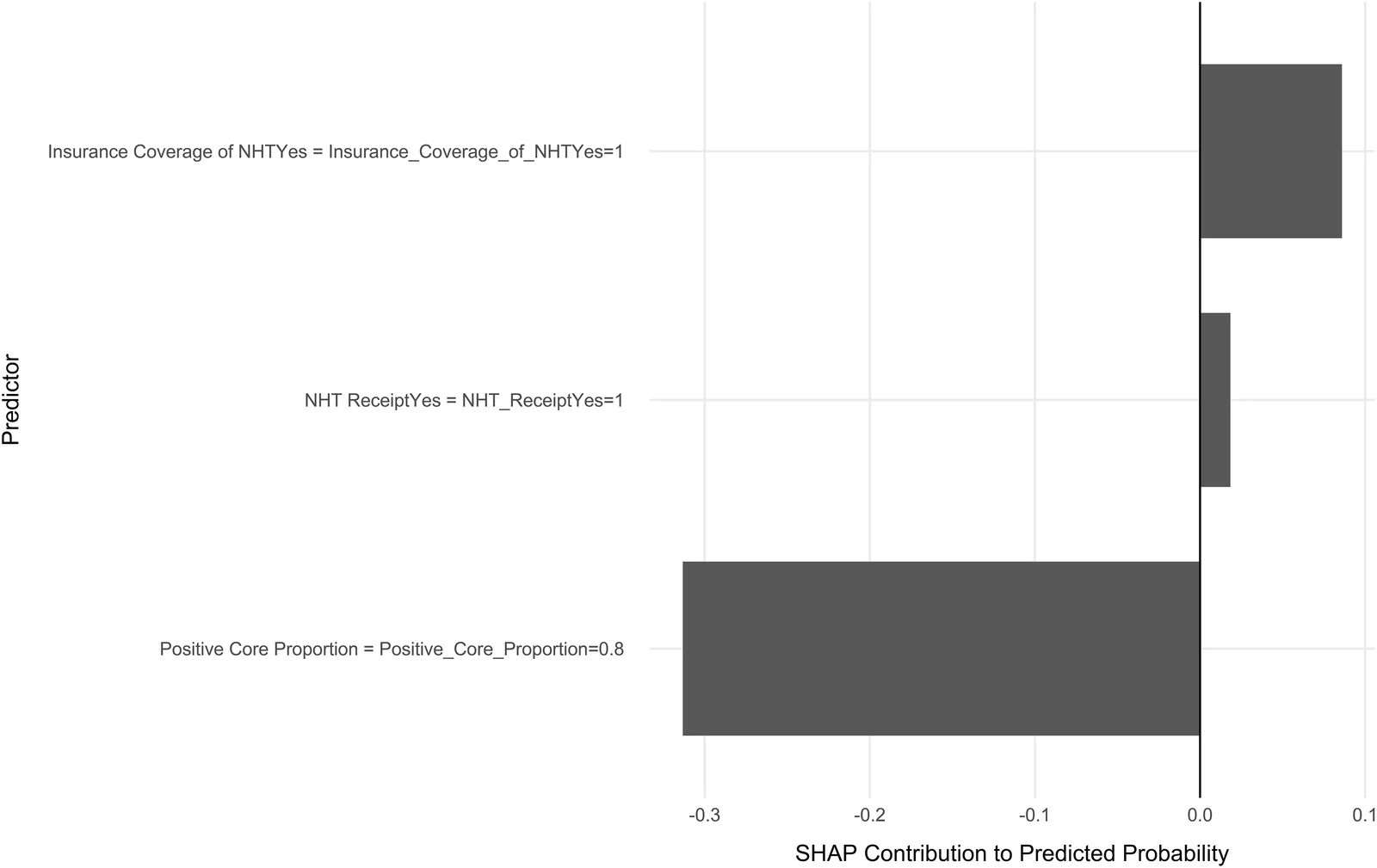
Local SHAP explanation for an individual patients.

Traditional Random Forest variable importance produced a ranking broadly consistent with the SHAP analysis, with insurance coverage remaining the highest-ranked predictor. Minor differences in the ordering of smoking history and positive core proportion were observed between the two methods, reflecting differences in the underlying measures of predictor importance. The comparison between Random Forest importance and SHAP rankings is presented in **Supplementary Figures 1**, **2**.

### Sensitivity analysis

To evaluate the influence of healthcare-access variables on model performance, a prespecified sensitivity analysis excluded insurance coverage for novel hormonal therapy from the candidate predictor set while maintaining the identical repeated nested cross-validation framework.

Removal of insurance coverage resulted in a modest reduction in Random Forest discrimination compared with the primary analysis, while overall classification performance remained similar. These findings suggest that insurance coverage contributed substantially to prediction of treatment intensification but was not solely responsible for model performance, indicating that additional clinical variables also contributed to treatment-selection decisions.

## Discussion

This study evaluated temporal trends in first-line systemic therapy for metastatic castration-sensitive prostate cancer (mCSPC) using national and institutional real-world data and explored factors associated with receipt of guideline-concordant treatment intensification through explainable machine learning. Three principal findings emerged. First, treatment intensification increased substantially over the study period at both the national level and within Avera Health, reflecting progressive adoption of contemporary evidence-based treatment recommendations. Second, repeated nested cross-validation demonstrated that several machine learning algorithms achieved comparable discrimination, although predictive performance was more modest and variable than initially observed using a single train-test split. Third, healthcare access and selected clinical characteristics, particularly insurance coverage for novel hormonal therapy, positive core proportion, and smoking history, were consistently identified as influential predictors of treatment intensification.

The treatment-pattern analysis demonstrated a progressive shift from androgen deprivation therapy (ADT) monotherapy toward novel hormonal therapy (NHT)-based treatment between 2018 and 2024 nationally and between 2018 and 2023 within Avera Health. These findings are consistent with the evolving treatment landscape following publication of landmark randomized clinical trials demonstrating improved overall survival with treatment intensification using docetaxel, androgen receptor pathway inhibitors, and selected triplet therapy regimens. The observed institutional trends suggest increasing implementation of guideline-concordant care over time and mirror broader national patterns reported in real-world observational studies.

Despite increasing adoption of treatment intensification, variation in treatment selection persists in routine clinical practice. Clinical decision-making for patients with mCSPC extends beyond disease characteristics alone and frequently incorporates patient preferences, comorbidity, anticipated treatment-related toxicity, medication availability, financial considerations, and healthcare access. Consequently, real-world treatment decisions often differ from those observed in randomized clinical trials, underscoring the importance of evaluating treatment patterns within routine clinical practice.

The exploratory machine learning analysis identified insurance coverage for novel hormonal therapy as the most consistently selected predictor across repeated feature-selection procedures. This finding is clinically plausible and aligns with previous real-world studies demonstrating that insurance status, medication access, and financial barriers influence adoption of intensified systemic therapy for patients with advanced prostate cancer. Insurance coverage likely reflects the ability to obtain timely access to newer systemic therapies rather than representing a direct biological determinant of treatment selection.

Positive core proportion and smoking history were also retained in the final explanatory Random Forest model. Positive core proportion may reflect disease burden and perceived disease aggressiveness at diagnosis, potentially influencing clinician willingness to recommend intensified treatment. Smoking history may represent broader differences in overall health status, comorbidity, or healthcare utilization rather than a direct determinant of treatment selection. Because of the exploratory nature of the present analysis and the limited cohort size, these findings should be interpreted as hypothesis-generating and require validation in larger multicenter populations.

An important methodological finding of the present study was the difference between results obtained using the original single train-test split and those obtained using repeated nested cross-validation. The repeated nested validation framework produced more conservative estimates of predictive performance and demonstrated substantial variability across validation resamples. This observation emphasizes the potential for optimistic performance estimates when machine learning models are evaluated using a single random partition, particularly in small datasets. By performing recursive feature elimination within outer training folds, restricting SMOTE to model-training resamples, and estimating performance across 100 independent outer validation resamples, the revised analytical framework reduced the likelihood of information leakage and provided a more realistic assessment of model generalizability.

Although the Decision Tree demonstrated the highest patient-level area under the receiver operating characteristic curve during repeated nested cross-validation, differences between models were modest and should not be interpreted as definitive evidence of superiority. Random Forest was selected for explainability analysis because it combined competitive predictive performance with a flexible and well-established framework for SHapley Additive exPlanations (SHAP). The broad agreement between SHAP-derived rankings and conventional Random Forest variable importance provides additional confidence that the identified predictors consistently contributed to model predictions despite differences in the underlying importance metrics.

The present study has several strengths. National treatment-pattern analysis were complemented by detailed institutional electronic health record review, allowing comparison of broader treatment trends with patient-level exploratory modeling. The revised analytical framework incorporated repeated nested cross-validation, leakage-aware feature selection, and resampling procedures designed to reduce optimistic bias. Furthermore, integration of SHAP improved transparency by allowing interpretation of predictor contributions rather than relying solely on conventional variable importance measures.

Several limitations should also be considered. The institutional cohort was small and derived from a single healthcare system, limiting statistical power and external generalizability. Although repeated nested cross-validation provides a more rigorous internal validation framework than a single train-test split, it does not replace external validation using independent patient cohorts. The observational design also precludes causal inference, and unmeasured clinical, socioeconomic, and provider-level factors may have influenced treatment selection. Finally, because treatment intensification was defined using initiation of therapy within 90 days of ADT initiation, patients experiencing delays beyond this interval may have been classified differently despite ultimately receiving intensified treatment.

Overall, these findings demonstrate increasing adoption of guideline-concordant treatment intensification for mCSPC while highlighting the continued influence of healthcare-access and clinical factors on treatment selection. The explainable machine learning framework identified several candidate predictors associated with treatment intensification, but these results should be viewed as exploratory and hypothesis-generating. Future studies incorporating larger, multicenter cohorts with external validation will be necessary to determine whether these predictors can support clinical decision-making or identify modifiable barriers to equitable implementation of guideline-concordant care.

## Conclusion

Treatment intensification for metastatic castration-sensitive prostate cancer increased substantially over the study period at both the national level and within Avera Health, reflecting progressive adoption of contemporary evidence-based treatment recommendations. Nevertheless, variation in receipt of guideline-concordant therapy persisted, suggesting that both clinical and healthcare-access factors continue to influence treatment selection in routine practice.

Using a rigorous repeated nested cross-validation framework, this exploratory study identified insurance coverage for novel hormonal therapy, positive core proportion, and smoking history as important factors associated with receipt of treatment intensification. Although predictive performance was modest and variable across resampling iterations, the use of explainable machine learning provided clinically interpretable insights into factors contributing to treatment decisions.

These findings should be considered hypothesis-generating because of the limited sample size and single-center design. Larger multicenter studies with external validation are needed to confirm the identified predictors and to determine whether explainable machine learning can support identification of barriers to equitable implementation of guideline-concordant treatment for patients with metastatic castration-sensitive prostate cancer.

## Data Availability

The raw data supporting the conclusions of this article will be made available by the authors, without undue reservation.
